# Combatting Covid-19. Or, “All Persons Are Equal but Some Persons Are More Equal than Others?”

**DOI:** 10.1017/S096318012000095X

**Published:** 2020-11-30

**Authors:** John Harris

**Affiliations:** Professor Emeritus, University of Manchester, Visiting Professor in Bioethics, Department of Global Health and Social Medicine, Kings College London and Distinguished Research Fellow, Oxford Uehiro Centre for Practical Ethics. Faculty of Philosophy, University of Oxford, Oxford, UK

**Keywords:** Covid-19, vaccines, scarce resources, value of life, premature death, justice, equality, public health

## Abstract

Vaccines, when available, will prove to be crucial in the fight against Covid-19. All societies will face acute dilemmas in allocating scarce lifesaving resources in the form of vaccines for Covid-19. The author proposes The Value of Lives Principle as a just and workable plan for equitable and efficient access. After describing what the principle entails, the author contrasts the advantage of this approach with other current proposals such as the Fair Priority Model.

## Introduction

All societies will, hopefully very shortly for the sake of humanity, be faced with problems of allocating scarce lifesaving resources in the form of vaccines for Covid-19 as they become available following satisfactory toxicity data confirming their safety. Already a number of proposals are being made as to how this should be done. What is needed is and is so far lacking are proposals which equally respect the value of all human lives at risk. I propose what I hope will prove a just and workable plan for distribution of any Covid-19 vaccine.

## The Value of Lives Principle

*All of us who wish to go on living have something that each of us values equally, although for each it is different in character, for some a much richer prize than for others, and we none of us know its true extent. This thing is of course ‘the rest of our lives’. So long as we do not know the date of our deaths then for each of us the ‘rest of our lives’ is of indefinite duration. Whether we are 17 or 70, in perfect health or suffering from a terminal disease, we each have the rest of our lives to lead. So long as we each wish to live out the rest of our lives, however long that turns out to be, then if we do not deserve to die, we each suffer the same injustice if our wishes are deliberately frustrated and we are cut off prematurely.*^1^

And this is true of course whether the “cutting off” is by criminal murder or by the deliberate and unjust denial of lifesaving resources, including vaccines.^2^

*The most moral and the most honourable way of dealing with the difficulties and anomalies that remain is to try to ensure that we have sufficient resources to devote to postponing death, wherever and whenever we can, whether for long or for short periods, so that we do not have to choose between people invidiously.*^3^

To act consistently with this rather abstract formulation, we, “societies” that is, should surely follow The Value of Lives Principle,^4^ which will, in the case of Covid-19:

1. Aim at creating sufficient vaccine as quickly as possible for an entire population, whether consisting solely of citizens or not, and ensure swift and free distribution to all. While sufficient stocks of vaccine are being produced or assembled for all, we should…
2. Prioritize vaccinating the most vulnerable to Covid-19 until there is sufficient supply for all. Priorities would probably include: (1) healthcare, social care, and other essential workers, (2) those over 65, (3) all with the so-called “underlying health conditions,” which render them more vulnerable to death from Covid-19, (4) those unable to self-isolate, and (5) young children.

There is some evidence for the likely acceptability and feasibility of this principle, because this is close to what health officials in the UK are currently doing (and have in past years done) with the annual flu vaccine. This is reported, as of September 22, 2020, by BBC News,^5^ as follows:

***“Health officials are trying to make sure everyone who needs a flu vaccine this autumn gets one.** Thirty million people will be offered the vaccine - the UK’s largest flu-immunisation programme to date. The fear is the annual flu season will coincide with a coronavirus surge. **How can I get a flu jab?** The NHS offers the flu vaccine via your GP or your local pharmacy. This year, (2020) the **free vaccination** is being offered in England to:*

- *Adults aged 65 and over*
- *People with **some medical conditions**, including diabetes, heart failure and asthma*
- *People who were required to **shield from coronavirus** - and anyone they live with*
- *Pregnant women*
- *Children aged from 2 to 11*
- *Health and social care workers*
- *Health officials in Scotland, Northern Ireland and Wales are planning to cover similar groups.*
- *Later in the year, the flu vaccine may be given to people aged 50 to 64. But the NHS says anyone aged 50-64, and in an at-risk group, should "not delay" having the flu vaccine.*
- *Doctors also want to increase vaccination levels in the most deprived areas and among people from ethnic minorities.*
- *Many pharmacies will also offer the jab privately for about £20, although Boots has temporarily suspended bookings for anyone under the age of 65, to ensure its existing stock is prioritised for those at highest risk.”*

There seems therefore to be no mystery about how societies might go about protecting their populations, and I do not dissent from the items on this list, although, of course, there is room for further discussion about the order of priorities.

## The So-Called “Fair Priority Model”

A recent, rather different, proposal and one which directly rejects The Value of Lives Principle has been made by an influential international group of academics. The proposal seems to be both gratuitously and unethically selective, as we shall now see. In their paper, *An ethical framework for global vaccine allocation,* Emanuel et al.^6^ suggest:

*“The pandemic forces allocators to decide where a vaccine’s harm-reducing powers are most urgently needed. Three dimensions of harm are important. Are the harms irreversible? How devastating are they? And can they be compensated?*

*On these three dimensions, preventing death—especially premature death—is particularly urgent. Death is uniquely devastating, and those who die for want of vaccine cannot be compensated later on. Surveys further suggest popular agreement that a premature death that prevents someone’s exercising their skills or realizing their goals later in life is worse than a death later in life… Ethicists have similarly argued that preventing early deaths—deaths that are more prevalent in poorer countries—is both prudent and ethical.”*

However, apart from the title of their paper, there is remarkably little about “global vaccine allocation” at all. That is to say, allocation to and between countries and populations. What we mostly find are recommendations about how nation states and healthcare systems should themselves prioritize whatever allocation they receive or manage to acquire, among their own populations, in different phases of supply. These priorities also run counter to the most universally agreed and plausible principle for ensuring equality between people, namely ensuring that equal concern, respect, and protection are delivered to all, regardless of gender, race, color of skin, elapsed lifetime or life expectancy, and all the other “usual suspects,” which urge us to discriminate unjustly.

In response, Emanuel *et al*. might claim that the three metrics, which, despite the fact that they primarily deal with allocation between individuals, do feed back into recommending how a global vaccine stock should be allocated *between* countries. For example, someone, say, at WHO, might look at the three metrics and decide how many doses a country should receive based on the three metrics.^7^

Emanuel *et al*. explicitly disavow most attempts to control how individual societies distribute their allocation, whatever it may be, except in extreme cases.

*“But outside of extreme cases, withholding vaccines to enforce conditionality inflicts disproportionate burdens, making conditionality rarely appropriate.*”

The Emanuel *et al*. *“Fair Priority Model”* is, for all practical purposes, a model for individual access rather than global distribution.

## All Unwanted Deaths Are Premature

The authors quote, seemingly with approval, surveys that suggest there is *popular agreement that a premature death that prevents someone’s exercising their skills or realizing their goals later in life is worse than a death later in life.*

It is unclear whether Emmanuel et al. think that “popular agreement” is sufficient authority for their recommendations. However that may be, we are clearly being invited to approve and endorse this “popular agreement” about who should be a priority for allocation of whatever vaccine supplies “allocators” get and distribute, as if it were an agreed fact.

More significantly, their conception of what makes a death premature is absurd. It is obvious, in a way that surely requires no further explanation, that **all deaths, at whatsoever stage of life, “prevent the deceased from realizing their goals later in life,”** and indeed, from developing new goals (in the case of young children) or any goals at all. Death is the great “preventer” of further….well, anything at all, for the individual who dies! **All deaths of persons whose meaningful life**^8^ **might have been extended are premature deaths**, however mature in years, or short of life expectancy the individual may be.

There is of course another definition of a “premature death” that presupposes that there is an appropriate span of years to human life, a “fair innings,”^9^ and that a death short of that target is by definition premature. There is however no plausible, nor agreed target of years^10^ by which degrees of prematurity can be measured.

As an English judge, Mars Jones J. said, in a judgement delivered in 1986:

“*However gravely ill a man may be…he is entitled in our law to every hour…that God has granted him. That hour or hours may be the most precious and most important hours of a man’s life. There may be business to transact, gifts to be given, forgiveness to be made, 101 bits of unfinished business which have to be concluded.”*^11^

There is no coherent sense in which the premature deaths of older people are less to be lamented than the deaths of younger people. And whereas it may be true that some “[e]thicists have similarly argued that preventing early deaths—deaths that are more prevalent in poorer countries—is both prudent and ethical (10, 13),” many others, myself included, would reject any such claim… and for good reasons, which we now explore further.

## “Everybody Is to Count for One, Nobody for More than One”

While “preventing early deaths—deaths that are more prevalent in poorer countries—might seem both prudent and ethical,” it is not necessarily either prudent or ethical to systematically prevent early deaths *at the cost of later deaths for other individuals.* Particularly when both the early deaths appear to be much less likely to occur in the case of Covid-19 and in the absence of a plausible impartial principle of just distribution of rescue. It is “rescue” that is clearly at issue here. However, there is such a principle available. It is derived from Jeremy Bentham’s famous “dictum” that at the heart of both justice and democracy is the principle that “everybody is to count for one, nobody for more than one.” This is the only truly impartial principle of the value of life.^12^

Although implicit in his writings, Bentham’s “dictum” comes to us via “a guest appearance” in Mill’s essay “Utilitarianism.” This essay first appeared as a series of three articles published in *Fraser’s Magazine* in 1861, which were collected and reprinted as a single book in 1863.^13^

This principle can be more clearly understood to say that all persons matter equally, regardless of who or what they are, regardless of gender, race, color, nationality, religion, age, level of ability or disability, and all the rest, including vitally, health status, and life expectancy. Abandon this principle and we are all, individuals and societies, faced with a myriad of invidious and arbitrary choices to which there are no agreed solutions and no plausible ones neither. Moreover, Bentham’s dictum protects us from falling prey to those who think they can justify distinctions between the “value” of different individuals. Such distinctions are simply a recipe for abuse.

If we were to attempt to translate Bentham’s dictum into a principle for the allocation of public resources to healthcare today, we might do worse than the following: “The principal objective of a public healthcare system should be to protect the life and health of each citizen impartially and to offer beneficial healthcare on the basis of individual need, so that each has an equal chance of flourishing to the extent that their personal health status permits*.*”^14^

As Mars Jones insisted, people value particular events within their lives disproportionately to the time required to experience those events or others. Without having available the vast detail of each person’s life and their hopes and aspirations within that detail, we cannot hope to do justice between lives. The only sensible, decent, and moral alternative is to count each life for one and none for more than one, whatever the differences in age and in other quality of life considerations.

It is this outlook that explains why murder is always wrong, and wrong to the same degree, regardless of the age or health state of the victim. When you rob someone of life, you take from them, not only all they have, but also all they will ever have. This is a difference in degree so radical that it makes for a difference in the quality of the act. However, the wrongfulness consists in taking from them something that they do not want to lose, and usually dread losing, more than anything else that they value.

## Individual Life Years Are Not Individual Lives

Emanuel *et al*. recommend the following as part of their *Fair Priority Model*:

*In phase 1, we propose using Standard Expected Years of Life Lost (SEYLL) averted per dose of vaccine as the metric for premature death.*

*SEYLL has three major advantages. First**, it regards all deaths as important but earlier deaths as particularly important.***^15^ *Thus, it integrates the aims of limiting harm and of prioritizing the least advantaged…*

And they conclude:

*The Fair Priority Model is the best embodiment of the ethical values of limiting harms, benefiting the disadvantaged, and recognizing equal concern *(*PAGE 1310).*

But as with the notorious quality-adjusted life year (QALY), life years are not lives, and maximizing *SEYLL* life years saved is not the same as maximizing lives saved, or deaths postponed.^16^ Quite the opposite in many cases!

Emanuel *et al*. say nothing about the fact that vaccinating the young, given that deaths among children and young people from Covid-19 seem to be significantly fewer, proportionately, than deaths among the over 55s, is on their own account of priorities counterproductive. Children should, according to them, be a very low priority indeed in this context, saving only for the danger of their grandparents catching Covid-19 from them.^17^

The *British Medical Journal* recently published the following key message (among others) related to the dangers of mortality resulting from Covid-19 infection:

- *For the general population, the risk of catching and then dying from covid-19 during 16 weeks of the pandemic was equivalent to experiencing around 5 weeks extra “normal” risk for those over 55, decreasing steadily with age, to just 2 extra days for schoolchildren.*
- *For those over 55 who are infected with covid-19, the additional risk of dying is slightly more than the “normal” risk of death from all other causes over one year, and less for under 55s.*^18^

## There Is Only One Thing Wrong with Dying

We have noted that a premature death is the death of someone who wanted to go on living or who there is no reason to suppose would not have wanted to live and whose wish, or the value of whose life, could have been respected but wasn’t. It is a death that could be, could have been, averted and wasn’t.

So long as we want our lives to continue, so long as we have something to live for, each of us rationally wants for ourselves the chance of continued life, and we are likely to go on wanting it so long as the quality of that life is worth having, that is, does not make us think that death would have been/would be preferable. There is only one thing wrong with dying and that is doing it when you do not want to.^19^ All those who die when they do not want to and could have their lives saved are arguably equally adversely affected, and each dies a premature death.

If everybody (every person) is not to count for one and none for more than one, then when Emanuel *et al*. claim “*The Fair Priority Model is the best embodiment of the ethical values of limiting harms, benefiting the disadvantaged, and recognizing equal concern,*” they are playing fast and loose with the meaning of “equal concern.”

Perhaps they themselves have not noticed, but “recognizing equal concern” *tout court* begs a question: For whom is equal concern being recognized? They seem to be hoping that a plausible conception of “equal concern” is not actually equal concern for all persons, or even for any person. But equal concern for an acronym, possibly even for an acronym that has been turned into an algorithm, which programs computers to prioritize candidates for vaccination, is not equal concern for all persons.

It is persons, all persons, that are part of the “everybody” who are to “count for one and nobody for more than one.” It is persons who die prematurely or not, persons who suffer, and persons who have hopes and fears and rights, duties, and interests. It is persons who have children and friends and parents who love them and who also do not want them to die when their lives could be prolonged or their deaths postponed.

It is important to note that while Emanuel *et al*. pay lip service to the idea that helping individuals is at the heart of their endeavor, as in the following passage, their espousal of a principle of progressively disvaluing the lives of older people fatally undermines any claim to showing equal concern and respect to persons.

*“A fair distribution of emergency supplies ultimately aims at helping individuals: They are the ones who live or die, prosper or are impoverished. Some authoritarian countries may do an excellent job of distributing vaccine to minimize health, economic, and other harms. As long as individuals benefit, fair global distribution among countries should neither require that intranational distribution of a vaccine be perfectly just nor seek to punish unrelated injustices.”*^20^

Emmanuel *et al*., in advocating equal concern for *SEYLL,* rather than equal concern for all persons, are not respecting the principle of equal concern. Equal concern is not a value in itself. Equal concern for nonsense is still nonsense. Not much better is equal concern for *SEYLL.* An “*expected year of life lost”* is not a person, an embodied self-consciousness, at all, let alone an instance of someone who is part of, one of, everybody—the body politic, the moral community, and the community of persons. A *SEYLL* is not a rights holder, and it has no interests. The safety of the people is what matters, not an abstraction. A life year does not want to be lost or gained, it fears nothing, and it hopes for nothing. As *King Lear* famously and cruelly observed to his beloved daughter Cordelia: “Nothing will come of nothing.”^21^

Once the subject of rights and interests, hopes and fears, desires, and relationships disappears, and is replaced by an abstraction, there is no one left who matters, nothing left that matters. The door is opened to selectively weeding out the older members of society, or as with the QALY, those whose expected **quality** of life will be lower than others.^22^ Of course things, ideas, concepts, etc. can matter, but only to persons.

On any metric that claims to protect the vulnerable, the old are vulnerable. When Emanuel *et al*. *“propose using SEYLL averted per dose of vaccine as the metric for premature death,”* and then insist “*preventing death—especially premature death—is particularly urgent,”* they are recommending the allocation of Covid-19 vaccines, and hence of the best chance of survival, both on a highly controversial definition of “premature death,” which few will understand or accept, and on *SEYLL,* an algorithmic-like acronym calculation,^23^ which few recipients or nonrecipients of vaccines will understand. Even less will understand why they are not being treated as equals with all others at risk of Covid-19.

The arguments of Emanuel et al. are a distortion of any normal understanding of what it is to show equal concern and respect for all persons. **It is simply not ethical for health professionals, (nor anyone else) to take on themselves the job of determining what a fair share of life is and hence what a fair share of life-preserving, or safety-ensuring, resources are.**

Not dissimilar arguments to those of Emanuel *et al*. have been the victims of flirtation by two British agencies.

The British newspaper *The Daily Telegraph* recently reported:

*“The former health secretary Jeremy Hunt downed tools and “refused to play” when asked to make a decision on who lived and who died during a cross-government pandemic simulation just three years ahead of Covid-19.*

*The incident - in which the minister was asked to turn off the ventilators of 4,000 fictional patients - happened during a mock Cobra meeting on the first day of Exercise Cygnus, the secret war game staged in October 2016 to test the UK’s pandemic resilience.*^24^ *It goes to the heart of a row about a triage protocol that was published by the National Institute of Health and Care Excellence (NICE) at the start of the pandemic and which is said by some to have resulted in thousands of frail and elderly patients being denied hospital care - a claim the NHS and professional medical bodies firmly deny.”*^25^

Jeremy Hunt is to be applauded for refusing to countenance even the fictional sacrifice of possibly as many as 4,000 people to make way for 4,000 others, whom other “others” judge to be more “valuable.”

The UK Daily Telegraph further reports:

*Official guidance has been issued to NHS intensive care doctors on how to decide which coronavirus patients should get critical care.*

*The guidance was issued by National Institute for Health and Care Excellence (NICE) (on 22^nd^ March 2020) and provides an “algorithm” to “help” doctors decide who should be admitted to critical care and who should not. The NICE guidance does not categorise potential patients by age but instead asks doctors to score patients on a nine-point “clinical frailty scale” [CFS]. At one end of the scale, with a score of one, are the “Very Fit” - people who are “robust, active, energetic and motivated”, and who “exercise regularly”. At the other end, with a score of nine, are the “Terminally ill”.*

*The NICE algorithm divides patients at a score of five, the “Mildly Frail”. Those with a score of less than five who would like critical care are considered well enough to benefit, subject to a review of any underlying conditions and the severity of their illness. Those scoring over five are put through a process where doctors must decide if critical care is “considered appropriate” before proceeding.*^26^

I myself can see no reason why even any members of NICE, nor anyone else, should be “considered appropriate” for death postponing critical care above anyone else, whatever their score in their own algorithm. But then I admit to suffering from “metaphorical jaundice” (the worst kind!). It is a truism that bears repeating that lifesaving is just death postponing, and to save a life is simply to shift the moment of death further into the future.

In 1651, Hobbes famously insisted:

*The office of the sovereign, be it a monarch or an assembly, consisteth in the end for which he was trusted with the sovereign power, namely the procuration of the safety of the people; to which he is obliged by the law of nature.*^27^

For Hobbes, and for anyone committed to equal concern for all people, “the safety of the people” means **the safety of all the people**. And the first requirement of safety is preservation of the lives of those who wish to have their lives preserved. That is why The Value of Lives Principle is at the heart of both justice and, of her daughter, democracy.

This is the backbone of any social contract, actual or implied. Once those whom Emanuel et al. term “allocators”^28^ of lifesaving vaccines have the power to choose to whom they will offer safety, then the doors are wide-open to abuse. It is clear that Emanuel et al. are highly engaged by the necessity to rule out possible abuses of the distribution of Covid-19 vaccine, but they have chosen a methodology well-calculated to have the opposite effect. They have produced a recipe for weeding out some of the most vulnerable, namely the elderly who find themselves “beyond the Pale” in virtue of their stipulated allocation of more *SEYLL* if they are not saved*.* We are all surely entitled to equal consideration of the value of our lives, not the discounted value that the self-interest of others is prepared to award us, to protect their own lives.

## “ALL ANIMALS ARE EQUAL BUT SOME ANIMALS ARE MORE EQUAL THAN OTHERS”^29^

George Orwell made dramatically clear the fraudulent nature of this “commandment” in his story, *Animal Farm.* And the same is surely true of its analogous use, in “other words,” by Emanuel et al. They have failed to consider the immense insult that those whose lives are disvalued (and others like them) receive when they are told, or, more likely find out, that they have been eliminated from, or downgraded for, consideration for rescue, that their lives are “worthless” in the sense of “worth less” than those of others. No one, no person, should suffer insult in this way.

I use here the term “insult” to cover its medical meaning as “injury” or “damage,” and also its more common use in English to mean “some word or gesture that is calculated to demean a person in the eyes of others.”^30^

It is difficult to understand how any decent^31^ person could imagine that “*recognizing equal concern”* is compatible with the suggestion of Emanuel *et al*. that we should regard: “*all deaths as important but earlier deaths as particularly important.”* This is not only straight out of Orwell’s *Animal Farm,*^32^ but also a clear example of what Orwell, in his later book *Nineteen Eighty Four,* ridiculed as “Newspeak.” The Appendix to *Nineteen Eighty Four* provides a comprehensive guide to Newspeak,^33^ which affords a key to understanding the “sleight of hand,” which enables Emanuel *et al*. to depersonalize “equal concern” and to value units of lifetime as if they were persons.

In writing of Charles Dickens, Orwell notes that Dickens “had the vision to see that” a central problem for humanity is “how to prevent power from being abused.” And Orwell concludes: “‘If men would behave decently, the world would be decent’ is not such a platitude as it sounds.”^34^ *Animal Farm* is an essay about the abuse of power and of language and about the importance of “decency.” In particular, it exposes the dangers of thinking that the idea that some people are more equal than others can, even in exceptional circumstances, be defended. Even in such a situation as the Covid-19 pandemic, we must never abandon this fundamental principle.

## Notes

1. Harris J. The Value of Life. London, UK: Routledge; 1985, at 89; Chapter 5.

2. See my book, *Violence and Responsibility.* London, UK: Routledge and Kegan Paul;1980, now reissued in the series: Routledge Library Editions: Ethics, London and New York 2021 in which I argue this point in some detail.

3. Harris J. The Value of Life. London, UK: Routledge and Kegan Paul; 1985, at 110.

4. See Harris J. How to Be Good. Oxford, UK: Oxford University Press; 2016, at Chapter 11.

5. https://www.bbc.co.uk/news/health-53847025.

6. Emanuel EJ, Persad G, Kern A, Buchanan A, Fabre C, Halliday D, et al. An ethical framework for global vaccine allocation. Science. 2020 Sep 11;369(6509):1309–12.3288388410.1126/science.abe2803PMC8691258

7. Here I am particularly indebted to comments from Sadie Regmi.

8. A “meaningful life” is any life that can have meaning to the individual whose life it is. Meaning of course, in John Locke’s famous definition of a “person,” is impossible to any creature who is not “a thinking intelligent being that has reason and reflection and can consider itself the same thinking thing in different times and places…” Locke J. An Essay Concerning Human Understanding. In: Pringle-Pattison AS, ed. Oxford, UK: The Clarendon Press; 1964, at Book II; Chapter 27. Locke formulated this definition in the middle of the seventeenth century.

9. I considered the “fair innings argument” and indeed named it, in *The Value of Life*, op cit. pp. 91–4,101.

10. See Harris J. Clones, Genes and Immortality. 2nd ed. Wonderwoman and Superman. Oxford, UK: Oxford University Press; 1998, fully revised and updated; Harris J. Intimations of immortality – The ethics and justice of life extending therapies. In: Freeman M, ed. *Current Legal Problems.* Oxford, UK: Oxford University Press; 2002:65–97; Harris J. Intimations of immortality. *Science* 2000 Apr;288(5463):59.

11. *R v Carr, The Sunday Times*, London, November 30, 1986. It is not inconceivable that Mars Jones had been influenced by this passage in my book *The Value of Life,* which was published in London, the year before his judgement. See Harris J. The Value of Life. London, UK: Routledge and Kegan Paul; 1985, at 89.

12. For the record, I am talking here of the lives of “persons” a concept on which I have elaborated in many places. See *The Value of Life*, op cit. pp. 18–27. See also Harris J. Violence and Responsibility. London, UK: Routledge and Kegan Paul; 1980 and 2021, at Chapter 1.

13. MillJS. Utilitarianism. In: Robson JM, ed. The Collected Works of John Stuart Mill, Vol. X: Essays on Ethics, Religion and Society. Toronto and London: University of Toronto Press and Routledge & Kegan Paul; 1969, at 257 (first published 1861).

14. Harris J. *How to Be Good*, op cit*.* Chapter 11. See also Harris J. What is the good of health care? *Bioethics* 1996 Oct;**10**(4):269–92.

15. My emphasis. It is incredible that such a suggestion should be made after Orwell so successfully lampooned such invidious distinctions in his novel *Animal Farm*: “ALL ANIMALS ARE EQUAL BUT SOME ANIMALS ARE MORE EQUAL THAN OTHERS.”

16. Harris J. QALYfying The Value of Life. Journal of Medical Ethics 1987 Sep;13(3):117–23.366903610.1136/jme.13.3.117PMC1375658

17. See, for example, https://ourworldindata.org/mortality-risk-covid.

**18**. Use of “normal” risk to improve understanding of dangers of covid-19. *British Medical Journal* 2020;370. doi:https://doi.org/10.1136/bmj.m3259.

19. I first put the point in this way in my evidence to the UK Parliament’s Select Committee on the *Assisted Dying for the Terminally Ill Bill.* London: House of Commons; 2004 Sep 9.

20. Emanuel et al. *op cit.* under the heading “Objections Considered.”

21. Shakespeare W. King Lear. In: Proudfoot R, Thompson A, and Kastan DS, eds. The Arden Shakespeare. Walton-On-Thames: Thomas Nelson and Sons Ltd; 1998, at 633; Act 1. Scene 1. Line 90.

22. This author pleads guilty to being on the wrong side of 75, but in his defence can note that he has advocated these same principles in print for more than 45 years.

23. Harris J. The immoral machine. *Cambridge Quarterly of Healthcare Ethics* 2020;29:71–9. Harris J. Reading the minds of those who never lived. *Clinical Neuroethics* 2019 Oct;28(4). Harris J. Who owns my autonomous vehicle: Ethics and responsibility in artificial and human intelligence. *Cambridge Quarterly of Healthcare Ethics* 2018 Oct;27(4):500–609.

24. https://www.telegraph.co.uk/global-health/science-and-disease/jeremy-hunt-downed-26thtools-refused-play-asked-turn-ventilators/ (last accessed 26 Oct 2020).

25. The details of the guidance referred to can be found at https://www.telegraph.co.uk/global-health/science-and-disease/revealed-official-guidance-doctors-decide-coronavirus-patients/ (last accessed 26 Oct 2020).

26. See note 25.

27. Hobbes T. *Leviathan.* In: Oakshott M, ed. Oxford, UK: Basil Blackwell; 1960, at 219 (first published 1651). Part II. Chapter 30. I expand on the scope of Hobbes conception of the safety of the people in Chapter 11 of *How to Be Good,* op cit.

28. “O good, allocators are you all….” With apologies to Shakepeare. *Richard II* Act 4. Scene 1. “Bolingbroke: Go some of you. Convey him to the Tower.” Richard: “O good! Convey! Conveyers are you all, That rise thus nimbly by a true king’s fall.” Shakespeare W. King Richard II. In: Proudfoot R, Thompson A, and Kastan DS, eds. The Arden Shakespeare. Walton-On-Thames: Thomas Nelson and Sons Ltd; 1998 Oct 13:692. Act 4.1. Lines 317–19.

29. I have capitalized this heading in homage to George Orwell who printed in capitals the famous slogan for his book *Animal Farm*: “ALL ANIMALS ARE EQUAL BUT SOME ARE MORE EQUAL THAN OTHERS.” Orwell G. Animal Farm. London: Secker and Warburg; 1945, at 86. I am using the Penguin English Library Edition of this book.

30. This definition of these terms is my own, but it is consistent with common English usage.

31. “Decency” was an important moral category for Orwell. See https://www.theguardian.com/books/2003/jun/07/biography.georgeorwell (last accessed 13 Oct 2020).

32. Orwell G. Animal Farm. London: Secker and Warburg; 1945. Clover, the old cart-horse spoke: “…it appears to me that the wall looks different. Are the seven commandments the same as they used to be Benjamin? …Benjamin read out to her what was written on the wall. There was nothing there now except a single Commandment. It ran: ‘ALL ANIMALS ARE EQUAL BUT SOME ARE MORE EQUAL THAN OTHERS.’”

33. Orwell G. *Nineteen Eighty Four*, op cit*.…*The Appendix: “The principles of Newspeak” pp. 343–55 provides a comprehensive guide to this element of the methodology of Emanuel et al.

34. Essay on Charles Dickens. In: Orwell G, ed. Collected Essays. London, UK: Secker and Warburg; 1961, at 48.

